# A High-Fidelity Virtual Environment for the Study of Paranoia

**DOI:** 10.1155/2013/538185

**Published:** 2013-12-17

**Authors:** Matthew R. Broome, Eva Zányi, Thomas Hamborg, Elmedin Selmanovic, Silvester Czanner, Max Birchwood, Alan Chalmers, Swaran P. Singh

**Affiliations:** ^1^Division of Mental Health and Wellbeing, Warwick Medical School, University of Warwick, Coventry CV4 7AL, UK; ^2^Department of Psychiatry, University of Oxford, Oxford OX3 7JX, UK; ^3^Division of Health Sciences,Warwick Medical School, University of Warwick, Coventry CV4 7AL, UK; ^4^Visualisation Group, WMG, University of Warwick, Coventry CV4 7AL, UK; ^5^School of Computing, Mathematics and Digital Technology, MMU, Manchester M1 5GD, UK; ^6^Department of Psychology, University of Birmingham, Birmingham B15 2TT, UK

## Abstract

Psychotic disorders carry social and economic costs for sufferers and society. Recent evidence highlights the risk posed by urban upbringing and social deprivation in the genesis of paranoia and psychosis. Evidence based psychological interventions are often not offered because of a lack of therapists. Virtual reality (VR) environments have been used to treat mental health problems. VR may be a way of understanding the aetiological processes in psychosis and increasing psychotherapeutic resources for its treatment. We developed a high-fidelity virtual reality scenario of an urban street scene to test the hypothesis that virtual urban exposure is able to generate paranoia to a comparable or greater extent than scenarios using indoor scenes. Participants (*n* = 32) entered the VR scenario for four minutes, after which time their degree of paranoid ideation was assessed. We demonstrated that the virtual reality scenario was able to elicit paranoia in a nonclinical, healthy group and that an urban scene was more likely to lead to higher levels of paranoia than a virtual indoor environment. We suggest that this study offers evidence to support the role of exposure to factors in the urban environment in the genesis and maintenance of psychotic experiences and symptoms. The realistic high-fidelity street scene scenario may offer a useful tool for therapists.

## 1. Introduction

Psychosis, and psychotic disorders such as schizophrenia, is characterized by the presence of hallucinations (false perceptions) and delusions (false beliefs). It has become clear in recent years that the marked heterogeneity in the rates of schizophrenia and psychotic disorders across population groups [1] can be partly explained by urban birth and upbringing, migration, ethnicity, and what Cantor-Graae and Selten have termed “social defeat” [2, 3]. A particularly important recent body of research is the MRC AESOP study that demonstrated a twentyfold rate increase in the incidence of psychosis in London, compared with Nottingham and Bristol, and the very highest rates being within the Black and ethnic minority groups [4–8]. These epidemiological findings have strengthened continuum models of psychosis [9], which state that psychotic-like experiences are distributed in a normal nonclinical population and might be dependent upon many of the same variables as causally responsible for cases of the disorder [10]. Trying to connect psychological, biological, and social models of psychosis, and relating these to psychotic experiences and urbanicity in the general population, has become increasingly important to both clinicians and researchers [11].

In a series of remarkable studies, Myin-Germeys and other colleagues from Maastricht [12–16] demonstrated a relationship between psychotic experiences, stress, and “daily hassles,” and further, that this sensitivity was in part consequent upon the reactivity of the participants dopaminergic system and their history of life events. Ellet and colleagues showed how the experience of walking through a busy urban street increased anxiety levels, negative beliefs about others, and exaggerated reasoning biases linked to the formation and maintenance of delusions [17]. Hence for schizophrenia and other psychotic disorders, recent epidemiological work has demonstrated both the heterogeneity of incidence rates and the role the urban environment, being part of an ethnic minority group and/or being a migrant, plays in the rates of the disorder. Further, experimental data suggests that being in the urban environment has an immediate and measurable impact upon levels of paranoia in both healthy controls and patients.

This study seeks to build on these recent findings and experimentally create an urban social environment where risk variables of interest can be carefully modified and controlled to test hypotheses as to the role the social environment and in particular the urban environment play in the generation of psychotic-like experiences, offering an experimental approach to examine social variables relevant to the generation of psychosis. Further, such a virtual environment may serve as a useful tool in the delivery of cognitive behavioural therapy (CBT) with patients with paranoia: the virtual environment serves as a safe, replicable place for behavioural experiments that can be manipulated and utilized by the patient remotely from the therapist. Virtual reality has been used as an important tool to study the aetiology of paranoia but thus far the scenarios developed have been those of a library and of a London underground train [18–20], both enclosed, internal spaces. With these studies, the focus has been on the “internal” psychological variables of the participants and how they predict paranoia. Our concern here is with environmental “external” variables, such as crowd density, traffic, and the nature of the buildings and street planning that may elicit paranoia and hence an urban street scene offers an environment whereby several features and variables can be manipulated independently and hence the role in the genesis of paranoia determined. Following the data outlined above, we would predict that a virtual urban experience would lead to higher levels of paranoia than a scenario in which the participants travelled on a virtual underground train.

Our aims and hypotheses in this study were as follows: firstly, to create a virtual reality scenario, using multimodal sensory inputs, that simulated a busy deprived inner city street scene; as such, this study was initially a feasibility and proof of concept study; secondly, to examine if exposure to this virtual environment evoked paranoid thoughts, such as believing that an element of the urban experience, for example, an avatar, posed a threat to them. Our hypothesis was that the urban street experience may be more likely to produce paranoia than that reported in published data utilizing an existing scenario used to evoke paranoia and also may be more useful for research examining social causes of psychosis and for developing adjunctive strategies to psychological interventions for psychosis.

## 2. Methods

### 2.1. Participants

The study was approved by the University of Warwick Ethics committee and participants were recruited from amongst the undergraduate and postgraduate students. Recruitment for the study was carried out by leaflet distribution, posters on campus, e-mails, and Internet webpage recruitment site. Participants were excluded from the study if they had a physical or sensory disability that would prohibit them from using the virtual reality equipment, if there was a history of psychiatric or neurological illness, or if they were dependent on alcohol or illicit drugs. All participants were blind to the hypotheses of the study.

Participants provided informed written consent. All testing, interviews, and exposure to the virtual reality scenario took place at the Warwick Manufacturing Group (WMG) International Digital Lab at the University of Warwick.

### 2.2. Scenario Design, Presentation, and Technical Specifications

#### 2.2.1. Technology

**Figure 1:**
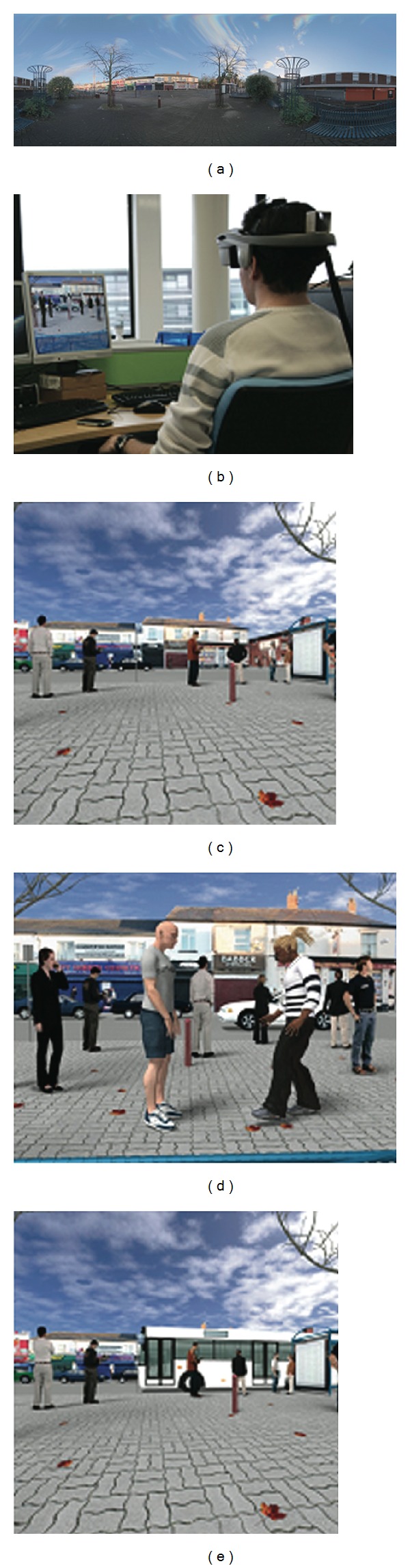
(a) Panoramic image of bus stop in Handsworth, (b) system set-up, (c) background scene, (d) avatars of young men in the scene, and (e) bus arrives.

The system comprised a NVIS nVisor SX head mounted display (HMD) and a Polhemus PATRIOT 6DOF Motion Tracking System controlled by an Intel Q6600 2.40 GHz Core 2 Quad with 4 GB of 800 MHz RAM and an NVIDIA GeForce 8800 GTX graphics card; see Figure 1. The HMD has SXGA resolution (1280 × 1024, 24-bit per eye), 60° diagonal field of view with 100% overlap, refresh rate of 60 Hz, and stereo headphones. It weighs approximately 1 kg and is connected to the PC via 2 × VGA cables.

#### 2.2.2. Scenario

The virtual reality environment used is a highly realistic multimodal (graphics and audio) virtual urban setting based on an actual street in the deprived area of Handsworth, Birmingham (UK) [21], including both the environment and virtual people (avatars). See Figure 1. In the scenario, the user of the system is sitting at the bus stop waiting for a bus. The environment was built using C++ with the OGRE game engine and a high dynamic range (HDR) panoramic image captured at the real bus stop using a Spheron SpheroCam HDR. Pouzar and Autodesk Maya software packages were used for the modelling and animations. The animation lasts for 4 minutes:

 0:00–1:30 mins: background with people and cars,
 1:30–1:40 mins: young men arrive in the scene,
 1:40–3:30 mins: young men stand around at bus stop,
 3:30–4:00 mins: bus arrives and at the same time men leave,
 4:00: end of experiment.

### 2.3. Measures

Prior to being exposed to the virtual reality scenario, all participants took part in an interview and completed questionnaires with one of the research team (EZ). These questionnaires included the Depression Anxiety Stress Scale (DASS, [1]), the Green et al. Paranoid Thoughts Scale (G-PTS [22]), Cardiff Anomalous Perceptions Scale (CAPS [23]), the Social Avoidance and Distress Scale (SADS [24]), Penn State Worry Questionnaire (PSWQ, [25]), and the Interpersonal Sensitivity Scale [26].

Subsequent to completing these questionnaires, participants donned the virtual reality headmounted display and entered the scenario where they remained for 4 minutes. The information the subjects received was that they were going to see a virtual bus stop in Handsworth, Birmingham, and that they would be sitting on a bench in front of it. They were asked to wait at the bus stop until the bus arrived (Figure 1). At the end of the scenario, they were debriefed and completed the State Social Paranoia Scale (SSPS, [27]). This scale has 10 items related to persecutory thoughts; each rated on a 5-point Likert scale and is designed to measure state paranoia elicited after an experimental exposure. A score of 1 on each item signifies absence and hence only scores totalled of greater than 10 signify the presence of paranoia. In total the whole procedure lasted for about 1.5 hrs per subject.

### 2.4. Statistical Analysis

Primary outcome of the statistical evaluation of this study was participants' persecutory ideation ascertained by SSPS score. SSPS scores after exposure to the urban street scene were compared to data published by Freeman and colleagues [20] for their study with participants in a virtual underground tube train. Two different analyses were conducted. First, as in the prior study, the data were dichotomised into participants who experienced paranoia (defined as SSPS score greater than 10) and those participants who did not (SSPS score less than or equal to 10). A likelihood-ratio *χ*-square test was conducted to compare the odds for experiencing paranoia between the two studies. A second analysis, informing about differences in the trend of score values, compared SSPS scores from both studies using six ordinal categories devised by Freeman et al. [20], SPSS scores < 10, 11–15, 16–20, 21–25, 26–30, >30. The categories are shown in Table 3. The comparison was performed employing an ordinal logistic regression model in which SSPS score is the outcome variable and study is the independent predictor.

Furthermore an exploratory analysis investigating the association of SSPS score (outcome) with demographic variables and pre-VR measures (predictors) was conducted. Adjusting for age and eye problems, an ordinal logistic regression model was fitted for each predictor separately. Estimates for predictors are presented as ordered odds ratios. The proportional odds assumption has held for all analyses.

All analyses were performed using SAS version 9.22.

## 3. Results

### 3.1. Participants

We recruited 32 participants. The participants were predominantly male, in their mid-20s, single, and international students studying for a postgraduate qualification with students from the whole campus being represented (see Table 1).

### 3.2. Pre-VR Measures

Data was collected on all participants on the measures detailed above. The G-PTS generates two scores, one for persecutory ideas and one for social reference, and these have been presented independently.

There were no significant correlations between any of the baseline, pre-VR measures, or the demographic variables.

We were unable to determine any strong predictors of paranoid thinking. That is, none of the investigated baseline and pre-VR measures significantly improved the model fit of the ordinal logistic regression model when included as a predictor. Furthermore, only one variable category (being short sighted) was significantly different from an odds-ratio value of 1 (see Tables 2 and 3).

### 3.3. Post-VR Measures

See Table 4. A striking finding with the SSPS data is that 65.6% of the participants experienced some persecutory ideation whilst taking part in the virtual reality scenario. Further, the mean SSPS in our sample is higher than reported by Freeman and colleagues, as is the proportion of participants who experienced any degree of paranoia whatsoever.

As detailed above, we carried out two analyses comparing our data in the urban street scene with that of Freeman and colleagues [20] stemming from an underground tube train scenario. First the data were dichotomised into participants who experienced paranoia (SSPS score greater than 10) and those participants who did not. In the Warwick study 65.6% (21/32) experienced paranoia, whereas only 47.5% (95/200) of the participants experience paranoia in the Freeman et al. [20] study. The estimated odds-ratio value for experiencing paranoia is 2.11 (95% CI = (0.967; 4.605)) which implies that participants in the Warwick study are more than twice as likely to experience paranoia. There is moderate evidence (*P* = 0.055) against the null hypothesis that the odds of experiencing paranoia in the two scenarios are in fact identical.

The second analysis compares the SSPS scores from both studies using categories as shown in Table 4. Individual SSPS scores for Freeman et al. were not available. The estimated odds that a Warwick study participant is in a higher score category instead of the given category is (odds-ratio =) 3.649 (95% CI = (1.833; 7.266)) times the odds for this being the case for Freeman et al. [20] participants. A fairly substantial association exists, Warwick participants tend to experience higher levels of paranoia. The likelihood-ratio statistic for the null hypothesis that study affiliation has no effect on SSPS score is highly significant (*P* < 0.001) so that it can be concluded that there is a strong association between study and score, with Warwick participants being more likely than those reported by Freeman et al. [20] to achieve a higher score.

## 4. Discussion

This study demonstrates that we have been successful in producing a virtual reality street scene, specifically of an urban, deprived environment including pedestrians. Further, this scenario was effective in producing paranoid ideation in the participants with approximately two-thirds of the subjects reporting persecutory thoughts after exposure to the scenario.

In comparison to a large study of healthy volunteers using a scenario of riding on the London underground [20], our scenario produced paranoia in a greater proportion of the sample. In our sample, 65.6% (21/32) experienced paranoia, whereas in the other study the proportion was 47.5% (95/200). Further, the urban street scene evoked higher levels of paranoia than the underground train scenario previously reported in the literature. Both scenarios involved other people represented as avatars, but the scenario used in this study was an outdoor street scene with avatars acting as a group and approaching the participant and hence may be more able to evoke paranoia. However, it is worth bearing in mind that we have not been able to conduct a true experimental comparison of the two scenarios and that the sample reported in this study differs in several ways from that reported by Freeman and colleagues. Our participants were much younger (mean age of 26 years, as opposed to 37), they were all students, the bulk of our participants were non-UK nationals who had come to Warwick University to pursue their studies, and the quality of the virtual environment was marginally higher (realistic high dynamic range images for representing the environment rather than less realistic computer models). Further, we were unable to directly compare the two groups of participants in terms of their pre-VR measures due to these data not being reported in the paper detailing exposure to the London underground VR scenario. Hence, it may be possible that our participants may have started at a higher baseline of paranoid ideation. However, unlike the work of Freeman and colleagues, we did not find any baseline predictors of post-VR paranoia; this may be due to the smaller sample size or that the variance in paranoia elicited was largely due to the effect of the social environment. This in turn reflects the different research question our group is addressing: rather than using a benign VR environment (an underground tube journey, a library) and looking at “internal” psychological variables, we have focused on examining “external” environmental factors that may impact on paranoia. We believe this scenario and its future refinements allow a unique opportunity to experimentally test hypotheses that relate urbanicity to psychosis by allowing investigators to manipulate independently variables, for example, traffic and pedestrian density, and determine these variables' effect on levels of paranoia. Such work would aid in the development of studying both the aetiology and treatment of psychosis and psychotic disorders [28–31].

## 5. Conclusion

We suggest that this study offers further experimental evidence to support the role of exposure to factors common in the urban environment in the genesis and maintenance of psychotic experiences and symptoms. Further, the realistic high-fidelity street scene scenario may offer a useful tool for therapists in constructing behavioural experiments with their clients.

Future work will consider further how the fidelity of the computer simulation may affect the quality of the exposure. In particular, as part of investigating the role of urban variables, we will investigate the impact of additional modalities, such as smell, for example, of alcohol on young men's breath.

## Figures and Tables

**Table 1:** Demographic data on participants.

Age (mean, SD)	Gender (M : F)	UK nationality (%)	Relationship status	Years of higher education (mean, SD)	Status of degree being studied for undergraduate : postgraduate
25.9 (SD 4.2)	23 : 9	34.4%	78.1% single, 21.9% married or in relationship	5.7 (SD 2.7)	5 : 27

**Table 2:** Pre-VR measures including Green et al. [22] Paranoid Thoughts Scale (G-PTS), Interpersonal Sensitivity Scale (IPS), Social Avoidance and Distress Scale (SADS), Depression Anxiety Stress Scale (DASS), the Penn State Worry Questionnaire (PSWW), and Cardiff Anomalous Perceptions Scale (CAPS).

Descriptive statistics					
	*N*	Minimum	Maximum	Mean	Standard deviation
GPTS-social reference	32	16	71	31.4375	12.01058
GPTS-persecutory ideas	32	16	63	24.3437	11.51887
IPS_total	32	58	115	87.6250	13.82553
SADS	32	1	17	7.1875	4.79541
DASS	32	1	79	21.5312	17.99280
DASS-depression	32	0	18	5.594	4.931
DASS-anxiety	32	0	31	5.594	6.734
DASS-stress	32	0	32	10.313	8.608
PSWW	32	3	15	8.0625	3.68902
CAPS-total score	32	0	23	7.8125	6.18759

**Table 3:** Individual variables are adjusted (controlled) for gender and having eye problems, apart from gender and eye problem themselves. The ∗ symbol indicates statistical significance (*P* < 0.05).

Variable	Odds ratio	95% confidence interval
Gender		
Female	0.77	0.18–3.29
Eye problem		
No problem	—	—
Short sight	4.62*	1.03–20.83
Other problem	1.01	0.19–5.44
Age	0.99	0.85–1.15
Plays computer games		
Yes	1.46	0.30–7.14
Nationality		
Non-British	1.82	0.48–6.84
Ethnicity		
White	—	—
Asian	1.53	0.38–6.10
Other	1.89	0.28–12.82
Social reference (3G-PTS)	0.97	0.92–1.03
Persecutory ideas (3G-PTS)	0.97	0.91–1.03
Interpersonal sensitivity	1.02	0.97–1.07
CAPS	1.02	0.90–1.15
DASS	0.99	0.96–1.03
DASS-depression	0.97	0.86–1.10
DASS-anxiety	0.98	0.89–1.08
DASS-stress	0.98	0.91–1.06
PSWQ	0.98	0.77–1.25

**Table 4:** Post-VR State Social Paranoia Scale (SSPS), comparing results from the Warwick urban scenario with Freeman and colleagues' [20] London underground tube journey scenario.

SSPS (mean and score frequency)	Warwick VR scenario	Freeman et al. 2008 [20]
Mean (SD)	17.5 (7.9)	12.26 (4.8)
≤10	11/3234.4%	105/20052.5%
11–15	6/3218.8%	64/20032%
16–20	4/3212.5%	16/2008%
21–25	5/3215.6%	9/2004.5%
26–30	2/326.25%	3/2001.5%
>30	4/3212.5%	3/2001.5%
